# Health Economic Impact of Software-Assisted Brain MRI on Therapeutic Decision-Making and Outcomes of Relapsing-Remitting Multiple Sclerosis Patients—A Microsimulation Study

**DOI:** 10.3390/brainsci11121570

**Published:** 2021-11-27

**Authors:** Diana M. Sima, Giovanni Esposito, Wim Van Hecke, Annemie Ribbens, Guy Nagels, Dirk Smeets

**Affiliations:** 1 icometrix, 3012 Leuven, Belgium; giovanni.esposito@icometrix.com (G.E.); wim.vanhecke@icometrix.com (W.V.H.); annemie.ribbens@icometrix.com (A.R.); guy.nagels@vub.be (G.N.); dirk.smeets@icometrix.com (D.S.); 2 AI Supported Modelling in Clinical Sciences (AIMS), Vrije Universiteit Brussel, 1050 Brussels, Belgium; 3 Department of Engineering, University of Oxford, Oxford OX1 3PJ, UK

**Keywords:** relapsing-remitting multiple sclerosis (RRMS), magnetic resonance imaging (MRI), brain MRI analysis software, non-evidence of disease activity (NEDA), Markov model

## Abstract

Aim: To develop a microsimulation model to assess the potential health economic impact of software-assisted MRI in detecting disease activity or progression in relapsing-remitting multiple sclerosis (RRMS) patients. Methods: We develop a simulated decision analytical model based on a hypothetical cohort of RRMS patients to compare a baseline decision-making strategy in which only clinical evolution (relapses and disability progression) factors are used for therapy decisions in MS follow-up, with decision-making strategies involving MRI. In this context, we include comparisons with a visual radiologic assessment of lesion evolution, software-assisted lesion detection, and software-assisted brain volume loss estimation. The model simulates clinical (EDSS transitions, number of relapses) and subclinical (new lesions and brain volume loss) disease progression and activity, modulated by the efficacy profiles of different disease-modifying therapies (DMTs). The simulated decision-making process includes the possibility to escalate from a low efficacy DMT to a high efficacy DMT or to switch between high efficacy DMTs when disease activity is detected. We also consider potential error factors that may occur during decision making, such as incomplete detection of new lesions, or inexact computation of brain volume loss. Finally, differences between strategies in terms of the time spent on treatment while having undetected disease progression/activity, the impact on the patient’s quality of life, and costs associated with health status from a US perspective, are reported. Results: The average time with undetected disease progression while on low efficacy treatment is shortened significantly when using MRI, from around 3 years based on clinical criteria alone, to 2 when adding visual examination of MRI, and down to only 1 year with assistive software. Hence, faster escalation to a high efficacy DMT can be performed when MRI software is added to the radiological reading, which has positive effects in terms of health outcomes. The incremental utility shows average gains of 0.23 to 0.37 QALYs over 10 and 15 years, respectively, when using software-assisted MRI compared to clinical parameters only. Due to long-term health benefits, the average annual costs associated with health status are lower by $1500–$2200 per patient when employing MRI and assistive software. Conclusions: The health economic burden of MS is high. Using assistive MRI software to detect and quantify lesions and/or brain atrophy has a significant impact on the detection of disease activity, treatment decisions, health outcomes, utilities, and costs in patients with MS.

## 1. Introduction

There are almost 25,000 newly diagnosed Multiple Sclerosis (MS) cases in the US each year, and nearly 1 million people are living with MS in the US (Atlas of MS, 2020, www.atlasofms.org, accessed on 15 October 2021). Relapsing-remitting multiple sclerosis (RRMS) is the most prevalent type of MS at diagnosis, with about 85% of people with MS being initially diagnosed with relapsing MS and approximately three times more females than males (Atlas of MS, 2020, www.atlasofms.org, accessed on 15 October 2021).

When a person is diagnosed with MS, the therapy goal is to stop or slow down the natural course of disease evolution, while balancing at the same time an acceptable level of burden, risks of side effects, and costs. Currently, there are more than twenty FDA-approved disease-modifying therapies (DMTs) available for RRMS patients (nationalmssociety.org/Treating-MS/Medications, accessed on 15 October 2021). These are intended to reduce the disease burden, disease activity, and progression, but they do not cure the underlying disease. The current treatment guidelines state that any evidence of disease activity while on consistent treatment should prompt the consideration of an alternative regimen to optimize therapeutic benefit [1]. Therapy selection, either immediately after diagnosis or in further follow-up, is made on a case-by-case basis and depends on the perceived level of clinical and subclinical disease activity and progression.

Evidence of clinical disease activity and progression are new relapses and disability worsening, as often measured by the Kurtzke Expanded Disability Status Scale (EDSS). Subclinical disease activity and progression are evaluated on brain (and spinal cord) magnetic resonance imaging (MRI) scans, by measuring the number of new and enlarging lesions as well as brain atrophy [2]. Though relapses and EDSS are considered primary endpoints in pivotal clinical trials and are an important therapeutic target in MS, yearly MRI scans have become part of standard MS monitoring and are crucial for clinical decision-making. In addition, “silent” progression due to brain atrophy has been found to be associated with long term disability accumulation in patients without relapses, suggesting that the process that underlies secondary progressive MS likely begins far earlier than is generally recognized [3].

Though MRI measures of new and enlarging lesions and brain atrophy are essential for therapeutic decision-making in MS, the typical radiological report is qualitative and based on a visual assessment. Detecting and quantifying disease activity based on brain MRI scans visually is a difficult and tedious task, and it is known that around 24% of radiological reports of brain MRI scans contain discrepancies [4]. Fortunately, thanks to recent imaging artificial intelligence (AI) innovations, reliable regulatory cleared software solutions for MRI volumetry are being increasingly used in clinical practice to enhance radiological reporting [5]. This technology brings potential advantages in terms of enhanced sensitivity for detecting subclinical pathologic aspects, as well as increased reproducibility compared to visual radiological evaluation [4,6,7].

In this paper, we focus on the potential health economic impact of using brain MRI reading and analysis software during decision-making in MS. To this end, a novel approach is proposed, where a cohort of RRMS patients is simulated based on a hidden state of disease activity, which is assessed with different decision-making strategies. The impact of these decisions over time is evaluated in terms of health outcomes and costs.

Evaluating the health economic impact of treatment decisions in MS is very relevant, as, in the US, MS is the second most costly chronic condition (after congestive heart failure), with more than $4 million in total lifetime costs per patient [8,9,10]. With several MS therapies available, it has been shown that adopting a more personalized medicine in MS, including data-driven clinical decision-making, has the potential to increase the health impact of existing treatments by over 50%, and therefore significantly reduce the costs [11].

The present simulated decision analytical model compares a baseline decision-making strategy for RRMS follow-up, in which only clinical evolution (relapses and EDSS progression) factors are used, with decision-making strategies involving MRI actively. In addition, the health economic analysis is simulated in the case of visual inspection of MRIs vs. using assistive software that detects new lesions and estimates the rate of brain atrophy. We run the model on a simulated cohort of RRMS patients from the moment they are prescribed a low efficacy DMT and evaluate the impact of the therapeutic decision-making path on outcomes, health utilities, and related costs, thereby adopting the US healthcare perspective.

## 2. Materials and Methods

### 2.1. Model Structure

**Figure 1:**
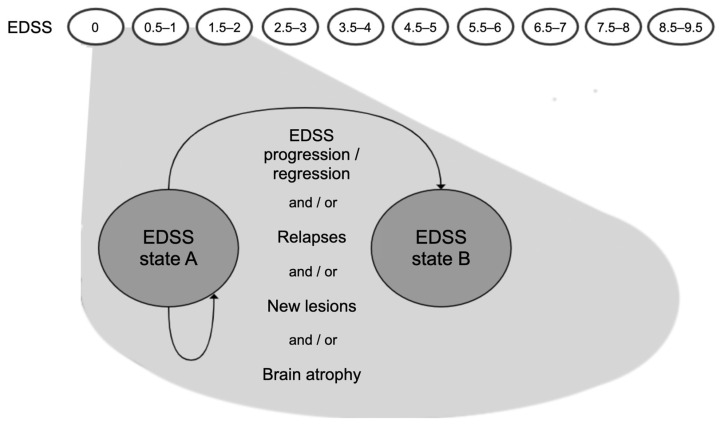
Schematic representation of the Markov model for disease progression; EDSS = Kurtzke Expanded Disability Status Scale; state A = EDSS state at the start of a model cycle; state B = EDSS state at the end of a model cycle. EDSS progression (B > A), regression (B < A) or stability (B = A) are possible. Clinical and subclinical disease activity may occur during each cycle.

We construct a decision-analytic model as a Markov model in which the states and state transitions are based on disability progression as measured with the EDSS, similar to previous widely used model structures of disability progression in RRMS [12,13,14]. In addition to these classical EDSS-based Markov models, the simulated patients can experience both clinical and subclinical disease activity, in the form of EDSS progression, relapses, new lesions, and/or brain atrophy; see Figure 1. The cycle duration is one year, which is the maximally recommended duration between neurological (including MRI) examinations for RRMS patients [15].

A hypothetical cohort of 1000 RRMS patients (female to male ratio 3:1) is simulated from the moment they start therapy on a low efficacy DMT. During each model cycle over a certain horizon (here, 10- or 15-years horizons are considered), patients can experience disease progression in terms of EDSS, relapses, lesion evolution, and/or brain volume loss. EDSS transition probabilities are defined based on historical data of natural disease progression in RRMS under the “best supportive care” [16], modulated by the efficacy of various DMTs in slowing down disease progression. The number of annual relapses (aR) and the annual number of new lesions (aNL) are randomly drawn from discrete probability distributions, constructed based on the same two factors: (1) natural history data of annualized relapse rates and new lesions in untreated “best supportive care” RRMS, and (2) the efficacy of various DMTs in suppressing relapses or new lesion formation, respectively. Brain volume loss is modeled as an annualized percentage of brain volume change (aPBVC), which is simulated from a Gaussian distribution with mean and standard deviation parameters that depend on DMT efficacy in slowing down brain atrophy. Details regarding all simulation parameters are presented in Section 2.3.

These 4 parameters characterize each patient during each cycle, and, by applying appropriate thresholds (defined in Table 1 in Section 2.2), these parameters define the hidden state of disease activity for each patient at the end of each model cycle. If any one of the 4 parameters exceeds its respective threshold, then we refer to the patient’s hidden state as “true disease activity”, which might signify a suboptimal response to therapy if the patient is on a DMT. Else, we label the patient as stable since there is no evidence of disease activity.

**Figure 2:**
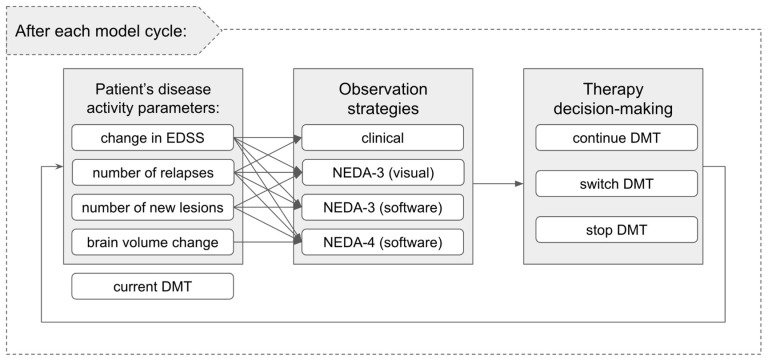
Schematic representation of the interaction between the hidden patient state, defined by the disease activity parameters, the considered observation strategies, and the therapy decision-making options after each model cycle.

Additionally, we consider a clinical observation model that simulates how a clinician reads and interprets the (partially) available disease activity/progression information, and how this translates into therapy decisions (see Figure 2). In this context, four different clinical decision strategies are compared:

1. clinical examination without MRI: disease activity or progression is established solely on clinical relapses and/or EDSS progression;
2. NEDA-3 (visual): clinical criteria (as above) are complemented by visually inspected MRI to detect lesion evolution;
3. NEDA-3 (software): NEDA-3 (visual) criteria as above are complemented by software-assisted lesion detection;
4. NEDA-4 (software): NEDA-3 (software) criteria as above are complemented by software-assisted brain volume loss computation.

**Figure 3:**
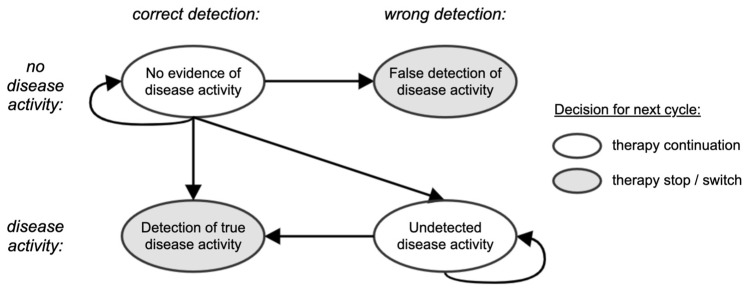
Schematic representation of the clinical decision-making process for a particular decision-making strategy. The disease activity status is either correctly or wrongly detected depending on the available information. At the next cycle, a patient stays on the same therapy if no disease activity is detected or can stop/switch therapy if disease activity is detected.

Under the considered observation strategies, not all parameters are available or are used. Thus, different observation strategies might lead to different therapeutic decisions. The options are to either continue the current DMT or to stop/switch it (see Figure 2 and Figure 3). For simplicity, we group DMTs in two families of “low efficacy DMTs” and “high efficacy DMTs”, where the grouping reflects differences in the efficacy profile [17]. All patients start on a low efficacy DMT, with the possibility to escalate to a high efficacy DMT.

### 2.2. Simulation of Observation and Therapy Decision Strategies

At the end of a model cycle, each therapy decision-making strategy is applied to each simulated patient. Table 1 presents the specific criteria defining detection of disease activity/progression for each strategy. The same thresholds are also applied for deciding the “ground truth” status based on the simulated hidden disease activity parameters. An increase in EDSS, the occurrence of relapses, the occurrence of new lesions, or abnormal brain volume loss, with the thresholds described in Table 1, thus indicate disease activity/progression.

For the EDSS and aR values, which are used identically in all strategies, it is assumed that the true values are available, without measurement error. Inter-rater variability and other uncertainties are not modeled for these clinical parameters. For aNL, it is known that visual detection of lesion activity on MRI follow-up scans can be imperfect, and is also highly dependent on radiologists’ experience and specialization [7,18]. Detection is significantly enhanced by assistive software, e.g., by tools that align MRI scans from different time points, or highlight new lesion candidates using a color code, with the rate of new lesion detection shown to be around 3–4 times higher when using assistive software compared to visual inspection of MRI scans [7,18,19,20,21].

In addition, visual MRI assessment is non-quantitative and precludes the use of a numerical threshold on the annualized brain volume loss, as this is impossible to assess with the naked eye. Specialized MRI volumetric software becomes a necessity to quantify subtle changes, which are typical of the order of −0.5%/year in MS patients [22]. However, aPBVC estimation with state-of-the-art software may suffer from measurement error of 0.1% (median absolute error) or higher, depending on the MRI machines, imaging sequences, use of different scanners for follow-up, etc [23]. In our model, we simulate measurement error on the aPBVC between two consecutive MRIs in the “NEDA-4 (software)” strategy by adding a zero-mean random error term on the ground truth aPBVC (see parameter choices in Table 1). However, when MRI scans are available for multiple years in a row, the brain volume loss computation would become more precise, because random measurement errors can be averaged out. To model this aspect, the standard deviation of the error term decreases in time with a factor equalling the square root of the number of available consecutive pairs of follow-up scans.

Based on these assumptions, true disease progression can be correctly or wrongly detected at the end of each cycle with any strategy, leading to a transition towards the “true detection of disease activity” or “undetected disease activity” states (i.e., the 2 bottom nodes in Figure 3), respectively. However, due to the nature of the simulation, only the “NEDA-4 (software)” strategy can lead to a wrong detection of disease activity (top-right node in Figure 3), which happens when there is no disease activity (i.e., all ground truth clinical and subclinical parameters correspond to a NEDA-4 status), but aPBVC gets slightly beyond the considered pathological threshold due to measurement error. In Section 3, we evaluate how often this happens, and what health economic consequences can be attributed to that. We also show the frequency of all other decision-making reasons per strategy and cycle.

Finally, if disease activity is detected based on the available parameters and the considered strategy, a patient on low efficacy DMT can switch to a high efficacy DMT. Whether the new DMT is successful or not is then evaluated at the end of the next model cycle under the same decision-making strategy.

If a patient with detected disease activity was already on high efficacy DMT, then a random switch within the high efficacy DMT family is allowed once, after which the patient is kept on the latest DMT until the end of the simulation or until it reaches EDSS greater than or equal to 7. In the latter case, it is assumed that the patient is taken off DMTs; the patient remains in the Markov model and follows a natural course of the disease but is ignored in the therapy decision-making model, meaning that no therapy decision state changes occur in the next model cycles.

### 2.3. Model Inputs—Simulation Details

#### 2.3.1. MS Disease Progression Parameters

As mentioned above, the hidden state parameters (EDSS, aR, aNL, and aPBVC) are simulated for each patient at each cycle. They are randomly sampled from appropriate probabilistic distributions, learned from untreated “best supportive care” RRMS natural history data, but modulated by relative efficacy gains characterizing each DMT family. Efficacy gains for EDSS progression and aR are taken from a network meta-analysis of 33 unique randomized trials with 21,768 patients presented in [17] evaluating more than 10 FDA-approved DMTs, which we recombine into 2 wide intervals corresponding to low and high efficacy DMT families (Table 2). To simulate how well a particular DMT suppresses disease activity in a particular simulated patient, a percentile score is randomly chosen for each patient on DMT and defines the efficacy gain factors for each hidden parameter. This percentile score stays in principle constant for each patient during model cycles until a change in DMT occurs for that patient. However, the model assumes that undetected patients with MRI activity experience a faster EDSS progression than stable patients. This is penalized by including an acceleration parameter (AP = 1.484) in the model to increase the probability of future progression prior to adjustment for the effect of DMT, as described in [24].

#### 2.3.2. EDSS

The disability states in the model are defined using steps 0 (normal) through 9.5 (helpless patient confined to bed and unable to communicate effectively or eat/swallow) of the EDSS. Each patient in the simulated cohort is initially assigned a random starting value for EDSS, uniformly sampled from 0 to 3 (Appendix A Table A1). In each model cycle, patients may stay in the same disability state, progress to a higher (worse) disability state, or regress to a lower (better) disability state. The unmodulated EDSS transition probability matrix is based on the British Columbia Multiple Sclerosis longitudinal observational cohort [16,25] and is presented in Appendix A Table A2. This “natural course” EDSS transition probability matrix is based on a mixed-sex cohort, therefore it is first modified for each patient by a sex-specific risk factor (1.05 for males and 0.97 for females, corresponding to an increased chance of EDSS progression in males, as observed in an analysis of the MSBase Registry data [26]). Then it is further modified for each patient by a relative risk factor based on the efficacy gain percentile score assigned to that particular patient and the assigned DMT:

- first, the relative risk factor is obtained from the interval corresponding to the patient’s DMT family (see Table 2, second column) assuming a uniform distribution and using the patient’s fixed percentile score;
- secondly, a new EDSS transition matrix is constructed by multiplying all transitions going from the patient’s current EDSS state towards states higher than the current EDSS state by f. For f < 1, this leads to less chance of EDSS progression. The remaining transition probabilities corresponding to EDSS states lower than or equal to the current state are scaled proportionally in order to ensure that all probabilities sum to 1 in each row.

At the end of a cycle, a new EDSS state is randomly generated based on the current EDSS state and the adapted transition probability matrix.

#### 2.3.3. Relapses and New Lesions

Both lesion activity and relapses are simulated as discrete random counts from zero-inflated distributions. The most widely accepted statistical model for annual counts of relapses or new lesions in MS is the negative binomial distribution, which is defined based on an average value μ and an over-dispersion parameter θ [27]. For aR, the mean μ is around 0.6–0.8 and varies with EDSS. We use estimates from [17], see Appendix A Table A3. The dispersion parameter is fixed at θ = 0.5. For aNL, we use experimental lesion count fitting results in untreated MS MRI datasets [28], to get approximate estimates for μ and θ as 10 and 0.5, respectively.

In order to simulate treatment effects on aR and aNL, the mean value is modulated by the efficacy improvement expected for low or high efficacy DMTs, respectively. Aban et al. [29], among others, argued that the dispersion parameter θ can be kept constant, regardless of the treatment, and only the mean μ should be modulated by the treatment effect as f * μ with f < 1. It remains to define the specific efficacy factors f for aR and aNL, respectively. For aR, there are various sources (including the meta-analysis in [17]) that provide these factors for a range of currently available DMTs for RRMS. Moreover, the patient’s sex is an additional modifier for the mean relapse rate, with the relapse frequency 17.7% higher in females compared with males [30]. We group the low and the high efficacy DMTs and express the efficacy gain in terms of a rate ratio (see Table 2, middle column). For the effect of DMTs on new lesions, we did not find efficacy gain estimates expressed similarly in the literature. However, we rely on the relationship between treatment effects on lesions and relapses uncovered in a comprehensive meta-analysis of MRI outcomes from 54 comparative randomized trials in more than 25,000 patients with RRMS [31]. The mean cumulative number of new or active T2 lesions, or gadolinium-enhancing lesions on monthly scans, counted over the follow-up period was extracted from each trial as the MRI endpoint for the analysis. The ratio between the average number of MRI lesions per patient in the experimental and the control groups was used to summarise the treatment effect on MRI lesions (lesions_effect) in each trial, and the effect on relapses (relapse_effect) was similarly computed. The treatment effect on lesions was found to be well correlated to the treatment effect on annual relapse rates, with log (relapse_effect) = 0.53 log (lesions_effect), R^2^ = 0.76. We apply this relationship to the rate ratios available from [17] for aR in order to obtain rate ratios for new lesions; see Table 2 (4th column).

#### 2.3.4. Brain Atrophy

Annual brain volume loss is simulated based on 2 Gaussian distributions that can be attributed to low efficacy and high efficacy DMT profiles. It is known that the distribution of aPBVC is highly overlapping between healthy subjects and untreated MS groups [22] and that brain volume loss is age-dependent [32]. High efficacy DMTs are able to bring the average annual volume loss down to values seen in healthy controls, while the distribution of brain atrophy rates in patients treated with low efficacy DMTs is significantly more pronounced and often at the same rates as in untreated MS [22,33,34]. Mean aPBVC values observed in the placebo arms and treatment arms across about a dozen MS clinical trials range from −0.43% to −0.78% for placebo, −0.44% to −0.60% for low efficacy DMT and −0.22% to −0.36% for high efficacy DMT (see [34] (Table 4)). Since we consider only two DMT families (low and high efficacy), the Gaussian model parameters used for aPBVC simulation in our model are taken from [22] and shown in Table 2 (right-most column).

**Figure A1:**
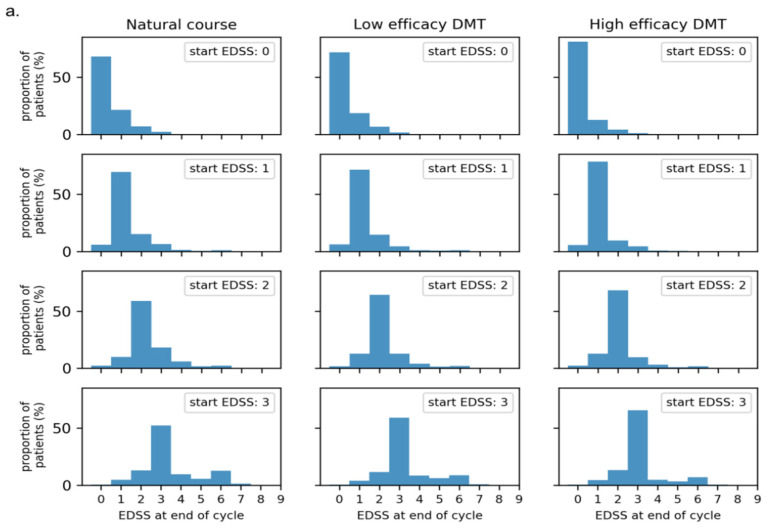

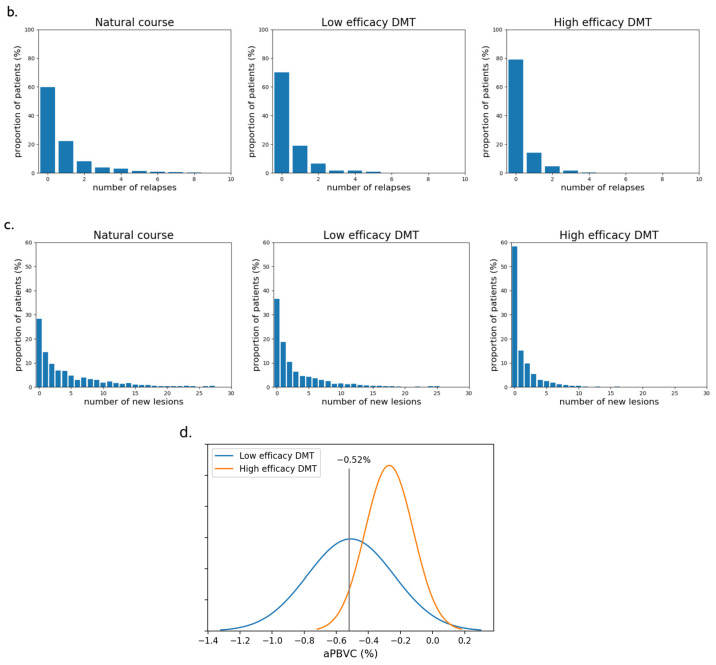
Distribution of simulated disease activity parameters. For comparison purposes, we show the untreated MS (natural course) distributions, together with those modulated by low or high efficacy DMTs. Efficacy rates are randomly generated to be representative for a whole family of DMTs, as specified in Table 2, and are thus not specific for a single DMT. (**a**) EDSS transitions showing the proportion of patients moving to the next EDSS state at the end of a cycle, when the EDSS at the beginning of the cycle was 0, 1, 2, or 3; (**b**) histograms of the number of relapses during one cycle, assuming an EDSS of 1; (**c**) histograms of the number of new lesions during one cycle; (**d**) Gaussian distributions of aPBVC for healthy controls, which is also used as target distribution for high efficacy DMT, and (untreated or on low efficacy DMT) MS patients. The −0.52% threshold giving 5% ‘false positives’ is illustrated (from [22]). With a mean of the MS distribution at −0.51%, the ‘sensitivity’ for this threshold is 49%.

The probabilistic distributions used to simulate aR, aNL, and aPBVC, as well as to perform EDSS state transitions, are illustrated in Appendix C Figure A1.

### 2.4. Outcome Measures, Utilities, and Costs

For each strategy, the total number of years (cycles) spent by each patient in the “undetected disease progression” state while on low efficacy DMT is computed. This number gives an indication about the potential time lost before escalating to a high efficacy DMT.

The simulation also keeps track of utilities and costs for each patient by assuming them to be conditional on the EDSS state and the number of relapses occurring in each model cycle. Mean utility (in QALYs) by EDSS state is sourced from [25] (see Appendix B Table A5) and ranges from 0.9248 at EDSS 0, which is close to the value 1 corresponding to perfect health, to a negative value of −0.2304 at EDSS 9 (a state that is subjectively deemed as being worse than dead). For each cycle spent in a certain EDSS state, the utility value corresponding to that EDSS state is added to the total utility of the simulation. Disutility values per relapse vary widely in the literature and are usually dependent on relapse severity. For simplicity, we consider only an average relapse disutility of −0.0437 per relapse as in [12,35]. No disutility is considered for the occurrence of new lesions or brain atrophy during a cycle. Based on these (dis)utility values, the annual QALYs averaged over the considered 10-years horizon can be computed for each strategy. As a 14.67-years horizon was used in [13] to evaluate the effect of DMTs, the QALY and cost analysis was also performed for 15 years.

The costs of conventional care due to disease progression, i.e., annual health state costs conditional on EDSS state, are taken from ([17] Table 20), see Appendix B Table A6. Also, an average cost per relapse of US$3069 is applied, cfr. [17,36]. All costs are inflated to 2021 US dollars and are allowed to vary by ±20% for each occurrence. In order to focus purely on costs driven by the patients’ health state, no DMT costs are explicitly included in the simulation, neither are costs for acquiring and reading MRI scans, or other factors such as adverse effects.

## 3. Results

### 3.1. Effect of Decision-Making Strategy on Detecting Disease Progression

**Figure 4:**
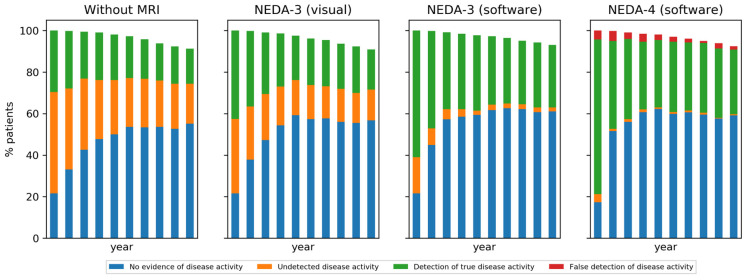
Comparison of decision-making strategies in terms of the proportion of patients in each model state according to the decision-making state transition model in Figure 2. Patients who reached EDSS 7 and are no longer on DMT are excluded.

Depending on the decision-making strategy, different proportions of patients having or not having disease activity at the end of each one-year cycle are observed. These proportions are illustrated per cycle in Figure 4 and reported as averages over the first 1, 5, and 10 cycles in Table 3. As expected, the proportion of patients with undetected disease activity decreases when increasing the complexity of the decision-making strategy, because there are more criteria that can lead to a detection of disease activity or progression. After the first model cycle, the simulation revealed 22% truly stable patients and 78% patients with disease activity. The 22% stable patients were correctly identified by all strategies, except for 4% wrongly perceived as having brain atrophy. However, among the 78% active/progressive patients, large proportions were missed by the clinical strategy without MRI (50%) and the NEDA-3 (visual) (36%). Averaged over more cycles, the two software-assisted strategies continue to take the lead in detecting more disease activity/progression compared to the clinical strategy without MRI and the NEDA-3 (visual) strategy. With the cumulation of more MRI follow-up data, the atrophy computation becomes less prone to measurement error, leading to a relatively low number (2%) of simulated decisions over the whole horizon that falsely indicates disease progression in the “NEDA-4 (software)” strategy. Since in our model all patients start on low efficacy DMT, there is more disease activity at the beginning of the simulation, and thus more chance to detect it, especially with the more sensitive strategies. Once patients switch to high efficacy DMT, the proportion of truly active patients, as well as those detected as such by the different strategies, decreases with time, until a stabilization takes place because only two switches are allowed in the high efficacy DMT family.

**Figure 5:**
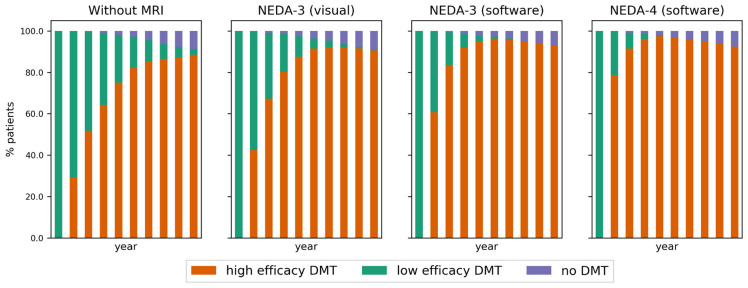
The proportion of patients for each strategy and cycle according to the family of DMT used. Patients who reached EDSS 7 are no longer on DMT.

As a consequence of detecting more patients with active disease in the first cycles of the simulation, a faster escalation to high efficacy DMT occurs in the decision-making strategies assisted by MRI. This is illustrated in the DMT distribution per strategy in Figure 5. Note the difference in escalation speed between the strategies.

### 3.2. Health Outcomes

On average, the simulation indicates that the considered cohort of RRMS patients stays on low efficacy DMT for:

- 3.2 ± 2.4 years for the clinical strategy without MRI,
- 2.3 ± 1.6 years for the NEDA-3 (visual) strategy,
- 1.7 ± 1.1 years for the NEDA-3 (software) strategy,
- 1.3 ± 0.7 years for the NEDA-4 (software) strategy.

While on this first-line therapy, the average time per patient in “undetected disease activity” state, which includes the year prior to the first decision moment, is:

- 2.8 ± 2.3 years for the clinical strategy without MRI,
- 1.9 ± 1.4 years for the NEDA-3 (visual) strategy,
- 1.3 ± 0.8 years for the NEDA-3 (software) strategy,
- 1.0 ± 0.2 years for the NEDA-4 (software) strategy.

There were no differences in these numbers when comparing the male and female subgroups, except for a mean difference of 0.1 years in detecting disease activity in males faster than in females with the clinical strategy; this can be attributed to the fact that there were proportionally more simulated male patients with EDSS progression than females. On the other hand, the slightly higher relapse rate in females did not influence these findings.

### 3.3. Utilities and Costs

Utilities per patient for each strategy are on average 6.48 to 6.71 QALYs over a 10-years horizon and 9.45 to 9.83 over a 15-years horizon for the different strategies (Table 4). The incremental comparisons between strategies in terms of the computed utilities presented in Table 4 indicate gains of up to 0.37 QALYs over the considered 15-years horizon compared to the clinical strategy without MRI.

The annualized costs per patient for each strategy over the considered 10-years and 15-years horizons, as well as incremental comparisons between the strategies, are presented in Table 5. These costs are driven by each patient’s health status (EDSS value per cycle) and disease activity (relapses per cycle). The maximal annual savings average is $2155 after 10 years and $2267 after 15 years when increasing the complexity of the decision-making strategy by adding both the MRI lesions and brain atrophy criteria to the clinical criteria.

## 4. Discussion and Conclusions

As many disease modifying treatments are available for MS patients, it is crucial to optimize therapeutic decision-making, especially as it has been shown that a more personalized medicine in MS has the potential to increase the health impact of existing treatments by over 50% [11]. In this context, a common paradigm is to aim for ‘no evidence of disease activity’ in each patient at each time point of evaluation. Depending on the definition, and the preference of the treating physician, disease activity is defined as a combination of relapses, disability (EDSS), new/enlarging lesions, and brain atrophy. However, it is known that, in a daily clinical routine setting, each of these has a measurement error as well as different sensitivities and specificities, resulting in a significant variation in decision-making.

The introduction of DMTs in the early nineties improved the lives of people with MS with about 0.5 QALYs accumulated in 15 years [13]. Our simulations (see Table 4) demonstrate that the introduction of a software-supported treatment paradigm (NEDA-4 with software) has the potential to add 0.34 QALYs compared to a visual analysis of brain MRI scans in 15 years, representing a relative improvement of 68% compared to the introduction of disease-modifying therapies.

To the best of our knowledge, this paper evaluates for the first time the effect of different treatment strategies in MS, as well as the availability of clinical and subclinical information in a microsimulation-based health economic setting. To this end, we modeled 1000 early MS patients, thereby simulating clinical and subclinical progression based on natural history data, modulated by treatment effect. These processes are assumed to be weakly correlated (e.g., the number of relapses per year differs across the EDSS spectrum; see Appendix A Table A3 for the employed mean rates), but the correlations are weak, because of high individual variability. We used a natural history EDSS transition matrix and applied a relative risk for each DMT family, in order to derive adapted transition probabilities between EDSS states. Furthermore, we included treatment effects in the simulation of relapse rates, new lesion rates, and brain atrophy rates. As the goal of this study is to evaluate current practice, rather than the future of more personalized decision-making, our model focused on assessing the time needed to detect disease progression or activity while on therapy under different decision-making strategies. DMT allocation and patient response to DMTs were simulated by assigning a DMT efficacy profile to each patient and it was assumed that the relative risk of EDSS transition, the rate ratio for relapse occurrence, the rate ratio for new lesion occurrence, and the annual brain volume loss were constant while the patient stayed on the same therapy, but changed (degraded) if the patient’s disease activity remained undetected and (potentially) improved if the patient was switched to another DMT.

The results of this paper indicate that using MRI and assistive software leads to benefits in terms of faster detection of disease activity and better long-term health outcomes due to faster escalation to high efficacy DMTs. Indeed, a clear difference in the detection of true disease activity was observed of 28%, 42%, 60%, and 74% after one year for decisions based on clinical information without MRI, NEDA-3 with visual MRI reading, NEDA-3 with software assistance, and NEDA-4 with software assistance, respectively. The undetected disease activity for these treatment strategies after year 1 is 50%, 36%, 18%, and 4%, respectively. As true disease activity that wasn’t picked up after one year is more likely to be picked up with a delay in the years later, and as we simulate that an increasing proportion of patients will be on a higher efficacy DMT throughout the years, the difference between the treatment strategies becomes smaller over time, with 20%, 25%, 35%, and 35% of detected true disease activity and 29%, 18%, 3%, and 1% of undetected disease activity, respectively, after 10 years. As a consequence, a higher proportion of patients would be switched to a higher efficacy DMT earlier when the NEDA-3 and NEDA-4 treatment strategies with assistive software are followed.

In MS, it is known that not being on the optimal treatment early in the disease has a significant health impact in later life and that being on a suboptimal treatment is similar to not being treated at all [11]. In this context, our results indicate that the average time a patient with MS has disease activity or progression while on treatment is 2.8 years, 1.9 years, 1.3 years, and 1.0 years when treatment decisions are based on clinical information without MRI, NEDA-3 with visual MRI reading, NEDA-3 with software assistance, and NEDA-4 with software assistance, respectively.

Regarding costs, the real total costs are composed of the sum of costs over the considered horizon, including costs of pharmaceuticals, medical visits, and indirect costs. Costs of DMT are typically treated separately from other health-related costs in health economics studies. Annual wholesale acquisition costs of individual DMTs in MS (see, e.g., ([17] Table 19)) in the US are high, at around $80,000 per year in 2021, but very similar between low and high efficacy DMTs. In our model, the proportion of patients stopping DMT would play a role in the overall average annual cost over the considered cohort. In this study, we decided to focus only on the costs associated with health states. The estimated costs driven by the patients’ health states show potential annual differences over the 10- or 15-year horizon of around $2200 per year on average. One has to take into consideration the cost of acquiring an annual MRI and performing radiological reading (with or without assistive software). Such costs can be in the range of $2000–$4000 in the US.

We aimed at developing our models as similar to clinical reality as possible, but theoretical models always have limitations. For example, therapy discontinuation was only modeled under the condition of reaching EDSS 7, while, in reality, intolerability, adverse effects, patient preference, and convenience play an important role in deciding therapy (dis)continuation. Taking such aspects into account would increase the percentage of patients that stop or switch DMTs, but this wasn’t modeled, as it would affect all treatment strategies considered in this study. In addition, our simulated observation model imposed a therapy switch if any of the observed parameters exceeded a predefined threshold, without taking into consideration the disease aggressiveness prior to DMT initiation. In practice, the thresholds for deciding that disease activity or progression occurs would need to be patient-specific rather than generic, to allow therapy continuation if there are potential benefits compared to stopping the DMT. In particular, deciding whether the rate of brain volume change is within normal limits or more pronounced compared to healthy controls should be done using age-specific thresholds and taking the stage of the disease into account, since the rate of brain atrophy is not constant over the life span or the course of MS [32,37].

Another way to refine the proposed microsimulation model is to incorporate additional confounding factors as part of the profile of each hypothetical patient. In particular, the age at MS onset could be included, since previous studies have shown that disability accumulates at a different pace depending on the onset age [38]: young-onset patients attain disability milestones earlier in life, but patients diagnosed later in life progress faster through the lower half of the EDSS scale. To account for such relations, the probabilistic distributions of the patient’s hidden state parameters, as well as the thresholds of the decision-making criteria, would require adaptations.

Though this study demonstrates a clear benefit in using assistive MRI software in the follow-up of MS patients, several design choices of the simulated model were not made in favor of using MRI (software). For example, the only parameter for which measurement error was generated was the annualized brain volume change. This led to a potential detection of false disease activity in 3% of the simulated decisions with the NEDA-4 (software) strategy over 10 years. This type of misdiagnosis deserves attention when balancing the advantages and disadvantages of implementing such a strategy in the real world. Furthermore, in this study, the EDSS state is used to measure progression, because it has historically been linked to costs and health outcomes. However, patients may have brain atrophy without changes in EDSS, but with a potentially large long-term impact on EDSS and/or cognition. Hence, more refined models and additional clinical evidence should be considered in future studies. Furthermore, in our model, inter-rater variability and other uncertainties were not modeled for the clinical parameters EDSS and the number of relapses. Nevertheless, it is known that estimating EDSS is prone to significant inter-rater variability and even daily variations.

In conclusion, using assistive MRI software to detect and quantify new lesions and/or brain atrophy has a significant impact on the detection of disease activity, treatment decisions, health outcomes, utilities, and costs in patients with MS.

## Figures and Tables

**Table 1:** Observation strategies and choice of decision parameters.

Strategy	Criteria for Detecting Disease Activity	Parameter Choices *
without MRI	at least n^relapse^ clinical relapses or EDSS disability progression **	n^relapse^: {**1**, 2, 3}
NEDA-3 (visual)	same as “without MRI” or at least n^lesion^ new lesions, but only a proportion p^lesion^ of true lesions are caught	n^lesion^: {1, 2, **3**, 4} p^lesion^: **33**%, 66%
NEDA-3 (software)	same as “NEDA-3 (visual)”	n^lesion^: {1, 2, **3**, 4} p^lesion^: 90%, **100**%
NEDA-4 (software)	same as “NEDA-3 (software)” or annualized whole brain volume loss > α% measurement error between two consecutive scans: ±ε%	α: {0.4, **0.52**, 0.72} ε: {0.1, **0.2**, 0.3}

* Default values used in the Section 3 are in bold; alternative parameter choices are discussed in Appendix C. ** EDSS disability progression is defined here as one of the following: if baseline EDSS 0, EDSS increase ≥ 1.5 points; if baseline EDSS ≥ 1, EDSS increase ≥ 1 point; if baseline EDSS > 5, EDSS increase ≥ 0.5 points. Abbreviations: EDSS, Expanded Disability Status Scale; NEDA, No Evidence of Disease Activity.

**Table 2:** Effectiveness of disease-modifying therapies on EDSS disability progression, relapses, new lesions, and brain volume loss.

Therapy Family	Relative Risk of Disability Progression ^c^	Rate Ratio for Relapse Rate ^c^	Rate Ratio for New Lesions ^d^	aPBVC ^e^
low efficacy ^a^	0.52–1.23	0.55–0.94	0.32–0.89	−0.51% ± 0.27%
high efficacy ^b^	0.25–0.90	0.22–0.63	0.06–0.42	−0.27% ± 0.15%

^a^ Beta interferon, glatiramer acetate, teriflunomide are included. ^b^ Alemtuzumab, natalizumab, ocrelizumab are included. ^c^ Values are pooled from [17], taking the minimum and maximum bounds from the intervals given for different DMTs in the 2 considered DMT families. See also Appendix A Table A4. ^d^ Values are estimated based on the relationship between the relative treatment effect on MRI lesions and the relative treatment effect on relapses, log (relapse_effect) = 0.53 log (lesions_effect) [31]; in other words, the values in columns 3 and 4 are linked through this equation. ^e^ Mean ± standard deviation of Gaussian distributions are taken from [22].

**Table 3:** The proportion of patients detected as stable or active in the four decision-making strategies. Results are reported for several model horizons, namely after the first cycle, and averaged over the first 5, and 10 cycles/years. No decisions are reported for patients who reached EDSS 7, but their respective proportions are listed for each strategy.

Year	Decision	Clinical without MRI	NEDA-3 (Visual)	NEDA-3 (Software)	NEDA-4 (Software)
1	stable	72%	58%	40%	22%
	- truly stable	22%	22%	22%	18%
	- undetected disease activity	50%	36%	18%	4%
	active	28%	42%	60%	78%
	- true disease activity	28%	42%	60%	74%
	- false detection of disease activity	-	-	-	4%
	reached EDSS 7	0%	0%	0%	0%
5	stable	74%	71%	60%	59%
	- truly stable	38%	50%	55%	58%
	- undetected disease activity	36%	21%	5%	1%
	active	24%	28%	39%	41%
	- true disease activity	24%	28%	39%	37%
	- false detection of disease activity	-	-	-	4%
	reached EDSS 7	2%	1%	1%	0%
10	stable	75%	72%	62%	60%
	- truly stable	46%	54%	59%	59%
	- undetected disease activity	29%	18%	3%	1%
	active	20%	25%	35%	38%
	- true disease activity	20%	25%	35%	35%
	- false detection of disease activity	-	-	-	3%
	reached EDSS 7	5%	3%	3%	2%

**Table 4:** Utilities (in QALYs) and incremental utilities per patient compared between strategies.

Strategy	Utility	Incremental Utility Compared to		
		Clinical without MRI	NEDA-3 (Visual)	NEDA-3 (Software)
over a 10-year horizon				
Clinical without MRI	6.48 ± 4.49	-	-	-
NEDA-3 (visual)	6.50 ± 4.63	0.03 ± 2.81	-	-
NEDA-3 (software)	6.67 ± 4.53	0.19 ± 2.80	0.16 ± 2.83	-
NEDA-4 (software)	6.71 ± 4.42	0.23 ± 2.79	0.20 ± 2.80	0.04 ± 2.81
over a 15-year horizon				
Clinical without MRI	9.45 ± 4.83	-	-	-
NEDA-3 (visual)	9.48 ± 4.97	0.03 ± 4.71	-	-
NEDA-3 (software)	9.78 ± 4.85	0.32 ± 4.68	0.29 ± 4.75	-
NEDA-4 (software)	9.83 ± 4.78	0.37 ± 4.63	0.34 ± 4.69	0.05 ± 4.67

**Table 5:** Annual costs related to health status (in US$ 2021) and incremental costs per patient compared between strategies.

Strategy	Cost	Incremental Cost Compared to		
		Clinical without MRI	NEDA-3 (Visual)	NEDA-3 (Software)
in a 10-years horizon				
Clinical without MRI	$33,809 ± $15,918	-	-	-
NEDA-3 (visual)	$33,176 ± $16,056	−$633 ± $18,606	-	-
NEDA-3 (software)	$32,272 ± $15,470	−$1538 ± $18,759	−$905 ± $18,686	-
NEDA-4 (software)	$31,655 ± $15,104	−$2155 ± $18,764	−$1521 ± $18,661	−$617 ± $18,384
in a 15-years horizon				
Clinical without MRI	$35,142 ± $17,304	-	-	-
NEDA-3 (visual)	$34,567 ± $17,437	−$576 ± $21,718	-	-
NEDA-3 (software)	$33,341 ± $16,565	−$1800 ± $21,366	−$1225 ± $21,348	-
NEDA-4 (software)	$32,875 ± $16,567	−$2267 ± $21,380	−$1691 ± $21,467	−$466 ± $20,831

## Data Availability

Not applicable.

## Appendix A. Model Inputs

Appendix ATable A1, Table A2, Table A3 and Table A4 present details relevant to the disease progression Markov model, including the considered initial distribution, state transition matrix, and assumed relations between variables.

**Table A1:** Initial EDSS state distribution of the RRMS population entering the model.

	EDSS 0	EDSS 1	EDSS 2	EDSS 3	EDSS 4	EDSS 5	EDSS 6	EDSS 7	EDSS 8	EDSS 9
Proportion	25	25	25	25	0	0	0	0	0	0

EDSS, Kurtzke Expanded Disability Status Scale.

**Table A2:** Transition Probability Matrix for 10-State Disability (EDSS) in the British Columbia Multiple Sclerosis Dataset.

Age ≥ 28 y		EDSS State at End of Year									
		0	1	2	3	4	5	6	7	8	9
EDSS State at Start of Year	0	0.6954	0.2029	0.0725	0.0217	0.0042	0.0014	0.0018	0.0001	0.00003	0.00000
	1	0.0583	0.6950	0.1578	0.0609	0.0164	0.0046	0.0064	0.0005	0.0001	0.00001
	2	0.0159	0.1213	0.6079	0.1680	0.0446	0.0185	0.0216	0.0017	0.0005	0.0000
	3	0.0059	0.0496	0.1201	0.5442	0.0911	0.0584	0.1165	0.0103	0.0035	0.0003
	4	0.0016	0.0221	0.0666	0.1152	0.4893	0.1039	0.1681	0.0258	0.0067	0.0006
	5	0.0005	0.0053	0.0294	0.0587	0.0874	0.4869	0.2731	0.0388	0.0188	0.0010
	6	0.0001	0.0013	0.0044	0.0250	0.0307	0.0408	0.7407	0.1090	0.0438	0.0042
	7	0.00001	0.0002	0.0005	0.0025	0.0073	0.0039	0.1168	0.6927	0.1606	0.0156
	8	0.0000	0.00001	0.0000	0.0003	0.0005	0.0005	0.0188	0.0557	0.9034	0.0207
	9	0.0000	0.0000	0.0000	0.00002	0.00004	0.00003	0.0018	0.0057	0.1741	0.8183

EDSS, Kurtzke Expanded Disability Status Scale. Source: [25].

**Table A3:** Annual Natural History Relapse Rate by EDSS Health State Used in the Model.

	EDSS 0	EDSS 1	EDSS 2	EDSS 3	EDSS 4	EDSS 5	EDSS 6	EDSS 7	EDSS 8	EDSS 9
Relapse rate *	0.71	0.73	0.68	0.72	0.71	0.59	0.49	0.51	0.51	0.51

EDSS, Kurtzke Expanded Disability Status Scale. * Mean number of relapses per patient per year. Source: ([17] Appendix Table E7).

**Table A4:** Treatment effect parameters for individual DMTs.

Treatment	Relative Risk EDSS Progression (Range)	Rate Ratio for Relapse Rate (Range)
Alemtuzumab	0.25–0.68	0.22–0.35
Dimethyl Fumarate	0.46–0.84	0.43–0.63
Fingolimod	0.51–0.90	0.39–0.55
Glatiramer acetate 20 mg	0.58–0.94	0.55–0.71
Interferon β-1a 30 mcg	0.63–1.00	0.74–0.94
Interferon β-1a 22 mcg	0.52–1.23	0.55–0.85
Interferon β-1a 44 mcg	0.52–0.99	0.54–0.73
Interferon β-1b 250 mcg	0.46–0.89	0.55–0.77
Natalizumab	0.37–0.84	0.25–0.40
Ocrelizumab	0.28–0.76	0.27–0.44
Peginterferon β-1a	0.37–1.02	0.47–0.86
Teriflunomide 7 mg	0.63–1.14	0.67–0.93
Teriflunomide 14 mg	0.52–0.97	0.56–0.79

Source: adapted from ([17] Appendix Table E7).

## Appendix B. Utilities and Costs

Appendix BTable A5 and Table A6 give numerical details about the cost-utility data used.

**Table A5:** Utility by EDSS state.

	EDSS 0	EDSS 1	EDSS 2	EDSS 3	EDSS 4	EDSS 5	EDSS 6	EDSS 7	EDSS 8	EDSS 9
Utility	0.9248	0.7614	0.6741	0.5643	0.5643	0.4906	0.4453	0.2686	0.0076	−0.2304

EDSS, Kurtzke Expanded Disability Status Scale. Source: [25].

**Table A6:** Annual costs by EDSS state (US$ 2021).

Annual Costs	EDSS 0	EDSS 1	EDSS 2	EDSS 3	EDSS 4	EDSS 5	EDSS 6	EDSS 7	EDSS 8	EDSS 9
Direct	$3221	$5536	$7851	$10,165	$12,481	$14,796	$17,111	$19,427	$21,741	$24,056
Indirect	$12,211	$16,704	$21,198	$25,692	$30,187	$34,681	$39,175	$43,669	$48,164	$52,658

EDSS, Kurtzke Expanded Disability Status Scale. Source: ([17] Table 20); values inflated to US$ 2021.

## Appendix C. Model Parameter Distributions

The probabilistic distributions used to simulate aR, aNL, and aPBVC, as well as to perform EDSS state transitions, are illustrated in Appendix C Figure A1 below.

The aR is shown for EDSS = 1 (mean aR is 0.58 for low efficacy DMT and 0.24 for high efficacy DMT), for EDSS = 2 (mean aR is 0.44 for low and 0.31 for high efficacy DMT), and for EDSS 3 (mean aR is 0.56 for low and 0.28 for high efficacy DMT). Mean aNL is 6.0 for low, and 2.3 for high efficacy DMT.

Appendix CTable A7 shows the effect of individual decision parameters on the proportion of simulated patients for which disease activity would be detected after one year, using the decision-making criteria in Table 1. 1000 simulated patients are generated for 3 situations: natural disease course, disease course modulated by low efficacy DMTs (with randomly generated efficacy according to the ranges given in Table 2 for the low efficacy DMTs family) and disease course modulated by high efficacy DMTs (with randomly generated efficacy according to the ranges given in Table 2 for the high efficacy DMTs family). For the number of relapses, the number of lesions, and aPBVC, several thresholds are considered, as listed in Table 1. These proportions do not show when several criteria are met simultaneously.

**Table A7:** The proportion of simulated patients where disease activity would be detected based on individual criteria is presented in Table 1, with different threshold choices.

	EDSS Progression	Relapse Progression Using n^relapse^ Threshold of			Lesion Progression Using n^lesion^ Threshold of				PBVC Progression Using α Threshold of		
	-	1 *	2	3	1	2	3 *	4	−0.40	−0.52 *	−0.72
natural course	22%	35%	17%	8%	64%	56%	48%	43%	66%	49%	21%
low efficacy DMT	19%	30%	12%	5%	64%	43%	38%	28%	66%	49%	21%
high efficacy DMT	12%	20%	6%	2%	48%	28%	15%	11%	19%	5%	0%

The * superscript indicates the selected threshold.
